# Time and space scattered volcanism of Mt. Etna driven by strike-slip tectonics

**DOI:** 10.1038/s41598-019-48550-1

**Published:** 2019-08-20

**Authors:** M. Firetto Carlino, D. Cavallaro, M. Coltelli, L. Cocchi, F. Zgur, D. Patanè

**Affiliations:** 1 Istituto Nazionale di Geofisica e Vulcanologia, Osservatorio Etneo, Catania, Italy; 2 Istituto Nazionale di Geofisica e Vulcanologia, Roma2, Roma, Italy; 3 Istituto Nazionale di Oceanografia e di Geofisica Sperimentale, Trieste, Italy

**Keywords:** Tectonics, Geophysics, Volcanology, Seismology, Geodynamics

## Abstract

High-resolution seismic reflection, magnetic and gravity data, acquired offshore of Etna volcano, provide a new insight to understanding the relationship between tectonics and spatial-temporal evolution of volcanism. The Timpe Plateau, a structural high pertaining to the Hyblean foreland domain, located offshore of southeastern Mt. Etna, is speckled by volcanics and strongly affected by strike-slip tectonics. Transpressive deformation produced a push-up and a remarkable shortening along WNW-ESE to NW-SE trending lineaments. Fault segments, bounding basinal areas, show evidence of positive tectonic inversion, suggesting a former transtensive phase. Transtensive tectonics favoured the emplacement of deep magmatic intrusive bodies and Plio-Quaternary scattered volcanics through releasing zones. The continuing of wrench tectonics along different shear zones led to the migration of transtensive regions in the Etna area and the positive inversion of the former ones, where new magma ascent was hampered. This process caused the shifting of volcanism firstly along the main WNW-ESE trending “Southern Etna Shear Zone”, then towards the Valle del Bove and finally up to the present-day stratovolcano.

## Introduction

Although Mt. Etna (eastern Sicily, Italy) provides one of the most intricate and studied examples of volcanism, its origin and spatial-temporal evolution are still the object of debate. Mt. Etna basaltic volcanism takes place in an atypical setting, i.e. on the front of the African vs European plates’ collisional belt and on the continental foreland crustal block, bounding to the west the subsiding Ionian plate.

Several geodynamic models were proposed to explain the origin of Etna’s magma in the mantle and its pathways in the crust, and the relatively fast shifting of the eruptive centres. The voluminous melting under Mt. Etna was related to the Ionian slab roll-back^1^, with magma rising through inherited faults, such as the northern portion of the Malta Escarpment system, which played the role of a dextral trans-tensional window^2,3^. A connection between Malta Escarpment structures and volcanism was also suggested by several authors^4–8^. Other models ascribe mantle upwelling to a regional crustal extension^9^ or to a localized hotspot-type mantle plume^10,11^. Finally, some authors^12^ inferred the primary role played by the transtensive reactivation of major offshore structures in Etna volcanism.

Volcanism associated with strike-slip tectonics is well-documented worldwide (e.g.^13,14^ and references therein); we suggest that migration of releasing and restraining zones along strike-slip faults^15,16^ may control the temporal and spatial distribution of volcanism.

**Figure 1:**
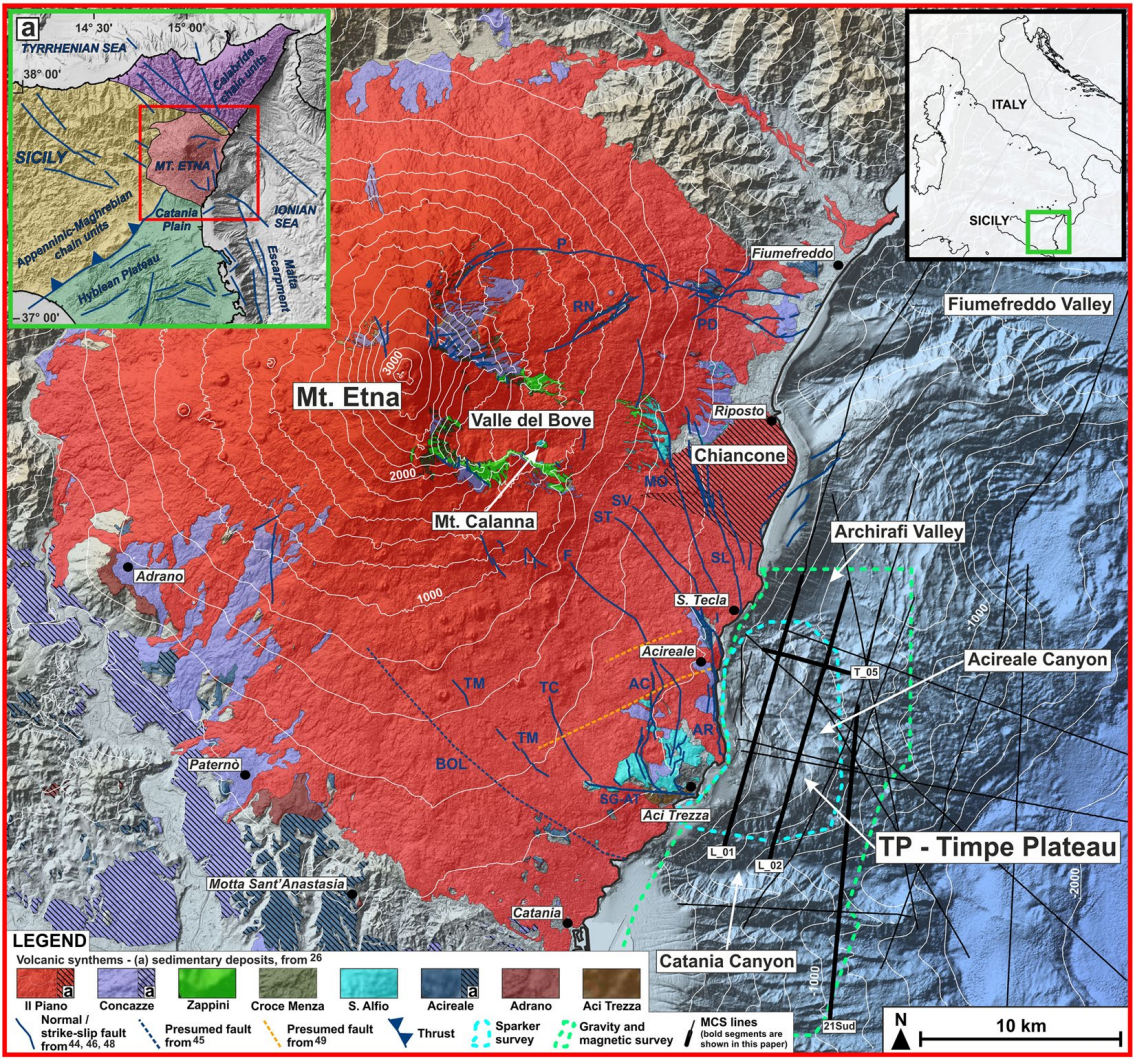
Morphological setting of the study area, volcano-tectonic features of Mt. Etna and geophysical dataset. Inset a - Geodynamic framework of eastern Sicily, modified from refs^19,28^. Main onshore faults (from refs^45,46,48,49^): BOL: Belpasso-Ognina Lineament; TM: Tremestieri; TC: Trecastagni; SG-AT: San Gregorio-Aci Trezza alignment; AC: Aci Catena; AR: Acireale; F: Fiandaca; ST: S. Tecla; SV: Santa Venerina; MO: Moscarello; SL: San Leonardello; RN: Ripe della Naca; PD: Piedimonte; P: Pernicana. Offshore faults and bathymetric data from ref.^44^ (Fig. 1 was created using the “CorelDraw X5” software).

In this paper, we studied the close correlation between the well-documented strike-slip tectonics affecting eastern Sicily^17–20^ and the evolution of Etna volcanism. The study is based on the interpretation of a large dataset of high-resolution single and multi-channel seismic reflection profiles, bathymetric, magnetic and gravity data, acquired offshore of the southeastern sector of Mt. Etna, where the oldest Etnean volcanics were found (Fig. 1), integrated with the geological and geophysical information onshore. We propose an innovative model to explain the evolution of this very active basaltic volcano at the front of a collisional belt, the rapid shifting of its eruptive vents and the occurrence of a local significant neo-tectonics.

Our findings also provide a striking advance in the wider understanding of tectono-magmatic interaction, giving a useful comparison for other volcanic regions worldwide.

### Tertiary volcanism in eastern Sicily

Tertiary volcanism in eastern Sicily started in the Tortonian, with tholeiitic-to-transitional alkaline basaltic eruptions in the northeastern sectors of the Hyblean Plateau, migrating northwards to the Catania Plain and offshore areas during middle-late Pleistocene^21–23^. This activity took place within the plates’ collisional region and is roughly coeval with the opening of the Tyrrhenian Basin^24^ (Fig. 1).

Since at least 500 ka, volcanism migrated further north, to the region of the present Mt. Etna^25^, where a significant shifting of the feeding systems occurred afterward, from the southern and coastal areas toward the Valle del Bove. This process likely reflects the rearrangements of the crustal setting in response to the regional tectonic regime^26^. Following the Geological Map of Etna Volcano^26^, the volcanic succession was subdivided into different synthems by means of a stratigraphic approach (Fig. 1).

First Etna’s volcanism began with the emplacement of tholeiitic to transitional^10^ subvolcanic bodies and eruptive products, currently exposed around Aci Trezza (*Aci Trezza Synthem*, 542 ± 86–496 ± 87 ka) and in the southwestern slope, between Motta S. Anastasia and Adrano (*Adrano Synthem*, 332 ± 43–320 ± 48 ka).

A hiatus of ca 100 ka separates this earlier scattered, fissure-type eruptive phase, from a later, more continuous Na-alkaline one^26,27^, suggesting a greater efficiency in magma ascent (*Acireale*, 180 ± 19–130 ± 5 ka and *S*. *Alfio*, 129 ± 8–112 ± 9 ka *Synthems*). Alkaline volcanics crop out along the Acireale coast, at the exit of the Valle del Bove (e.g. Mt. Calanna and along the Moscarello fault, MO, Fig. 1) and on the southern sector (between Aci Trezza and Adrano, Fig. 1).

Associated with the aforementioned phases, relicts of roughly N-S oriented eruptive fissures and dykes were found (e.g. the Motta S. Anastasia neck and dykes along Acireale coast^6,26^).

From at least 110 to ca 70 ka, the earliest central-type volcanoes occupied the area of the present Valle del Bove (*Croce Menza* and *Zappini Synthems*); a further northwestward shifting of the plumbing system led to the formation of the present bulk of Etna edifice (*Concazze*, 57-15 ka and *Il Piano Synthems* <15 ka)^25,26^.

### Regional geodynamic setting

Mt. Etna lies on top of the Apenninic-Maghrebian fold-and-thrust belt, overlaid by the Calabride chain units, and on the northern margin of the Upper-Cretaceous - Late-Miocene Hyblean carbonate succession (Fig. 1a), related to the African plate and flexuring northwards beneath the Plio-Pleistocene foredeep sediments^28^.

The Malta Escarpment fault system produces a prominent submarine scarp separating the Hyblean Plateau from the Ionian Basin, which is subducting northwards beneath the Calabrian Arc (Fig. 1a). The Malta Escarpment is related to a Mesozoic rifting, which led to the formation of the Ionian lithosphere, although its main morphological expression was produced later, during the Tortonian^7^. The northernmost portion of this fault system is characterized by NNW-SSE trending, east dipping, recent extensional fault segments, and related sedimentary basins, which have also been affected by a subsequent contractional deformation^7^.

The whole Hyblean Plateau is also dissected by NE-SW tectonic lineaments, active since the Messinian, and thought to be related to the foreland flexure beneath the thrust belt^29,30^. This tectonic setting formed several horsts and graben structures, recognized also offshore^23^, locally reactivated in reverse and left-lateral motion^31^, to absorb the ongoing collisional-related shortening^32^.

The interaction between the two aforementioned fault systems isolated several rhombohedral-shaped blocks along the Ionian coastal and nearshore areas of Hyblean Plateau^23,33^.

The northern sector of the Hyblean foreland is characterized by a northwestward motion, with an average velocity of 5 mm/a, with respect to the European frame^32^. Indeed, GPS measurements^34^ and earthquakes’ focal mechanisms^35^ point to a regional, roughly NW-SE oriented maximum horizontal stress axis, as a consequence of the plates’ convergence.

Moreover, a WNW-ESE to NW-SE trending fault system (South-Tyrrhenian System^36^) crosses the northeastern and central portion of Sicily^17–20^, accommodating, since the Tortonian^36^, the dextral displacement of the major tectonic domains caused by the roll-back of the Ionian slab and related spreading of the back-arc Tyrrhenian Basin^18,24^. The sinking slab is laterally torn from its continental margins, leading to a Subduction-Transform Edge Propagator (STEP) fault setting in northeastern Sicily down to the Ionian bathyal plain^7,12,19,37,38^.

The tectonic activity in this region is testified by recent significant seismicity (http://cnt.rm.ingv.it/) and historical large earthquakes (e.g. 1169, 1693 and 1990 earthquakes^30,39,40^).

### Neo-tectonics of Mt. Etna

Mt. Etna also shows evidence of local dynamics^41–45^, interpreted by some authors^41,42^ as due to the deformation induced by the ascent and emplacement of magma in the upper crust and the volcanic pile; this process favoured a fault-controlled gravitational sliding affecting the eastern flank of the volcano.

The coastal area between Aci Trezza and S. Tecla is cut by N-S to NNW-SSE trending, up to 8 km long, active extensional or dextral transtensive fault segments (e.g. Acireale and Aci Catena faults, AR and AC respectively, Fig. 1), pertaining to the Timpe fault system^46^. The latter was interpreted as the onland extension of the Malta Escarpment^4,5,47^ or related to the retrograde propagation of the continental margin instability^44^. The NW-SE trending Tremestieri and Trecastagni dextral transtensive faults (TM and TC, Fig. 1) affect the southern slope of Mt. Etna^46^.

All of these tectonic lineaments are sharply confined southwards by the WNW-ESE trending San Gregorio-Aci Trezza alignment (SG-AT, Fig. 1), characterized by a significant right-lateral motion^46,48^. To the north, the Acireale and Aci Catena faults terminate against the NW-SE oriented highly seismogenic Fiandaca (e.g. Mw 4.9, 26/12/2018 earthquake, http://cnt.rm.ingv.it/event/21285011/?tab = MeccanismoFocale#TDMTinfo) - S. Tecla - S. Venerina faults (F, ST, SV, Fig. 1), characterized by a right-lateral transtensive kinematics^46^. Other distinct NNW-SSE trending faults, pertaining to the Timpe system, extend northwards^46^ (e.g. Moscarello and S. Leonardello faults, MO and SL, Fig. 1). Along the major Timpe segments the products of a roughly coeval volcanism crop out^6,26^.

Mt. Etna region is also cut by NE-SW oriented normal faults, identified in the eastern slope (Ripe della Naca and Piedimonte faults, RN and PD^46^), around Acireale^49^ and offshore of Riposto^44^ (Fig. 1).

The widely investigated spreading of Mt. Etna eastern flank, which different authors link to volcano gravitational sliding^42,50,51^, with the contribution of shallow dyke intrusions^41,52^ or deeper plumbing system expansion^44^, is locally controlled by the above-described fault segments^43,45^. The unstable sector is bounded to the north by the WNW-ESE left-oblique Pernicana-Fiumefreddo fault system (P, Fig. 1), while to the south by a belt between the supposed Belpasso-Ognina Lineament, the Tremestieri-Trecastagni and the San Gregorio-Aci Trezza faults^43,45^ (respectively BOL, TM-TC and SG-AT, Fig. 1). Differential movements result in the northern sector spreading faster than the southern one, likely separated along the S. Tecla fault (ST, Fig. 1). Finally, the Valle del Bove slope failure^53^ testifies to the instability of Mt. Etna eastern flank.

Nevertheless, the Catania to S. Tecla coastal areas are undergoing a significant uplift, reaching 3 mm/a in the last 3.5 ka, decreasing northwards^54^, attributed to the migration of the Apenninic-Maghrebian fold-and-thrust belt front^55–57^.

Both regional and local tectonics also control the Mt. Etna offshore, which is characterized by a large, irregularly-shaped bulge, which projects eastwards with respect to the adjacent continental margins^44^ (Fig. 1) and affected by diffuse mass wasting processes^58–61^. The southern part of the bulge is characterized by the Timpe Plateau^61^ (TP, Fig. 1), interpreted, on the basis of morphological evidence, as the remnant of a primitive shield volcano^44^, whose products crop out inland along the NNW-SSE trending Acireale fault^6,25,26^ or alternatively as pertaining to the Hyblean foreland^59^.

## Results

### Morpho-bathymetric features

TP consists of a 75 km^2^ wide rhombohedral morphological high, extending for nearly 10 km offshore of the coast between Aci Trezza and S. Tecla along its N-S major axis (Fig. 1). TP is bounded to the east by a NNW-SSE scarp while its northern and southern margins are bounded by the Archirafi Valley and Catania Canyon, respectively^44,61^. The latter is characterized by a sinuous trend, which is different from the straight and narrow-spaced canyons affecting the continental margin to the south.

Acireale Canyon splits the TP into two sectors; the northern one, about 6 km wide, extends down to 650 m bsl, while the southern, nearly 7.5 km wide, deepens down to 1000 m bsl. TP displays an overall step-like arrangement with N-S to NW-SE high scarps and is also cut by other erosive features and affected by small and large-scale gravitational instability^58^.

### Magnetic data

**Figure 2:**
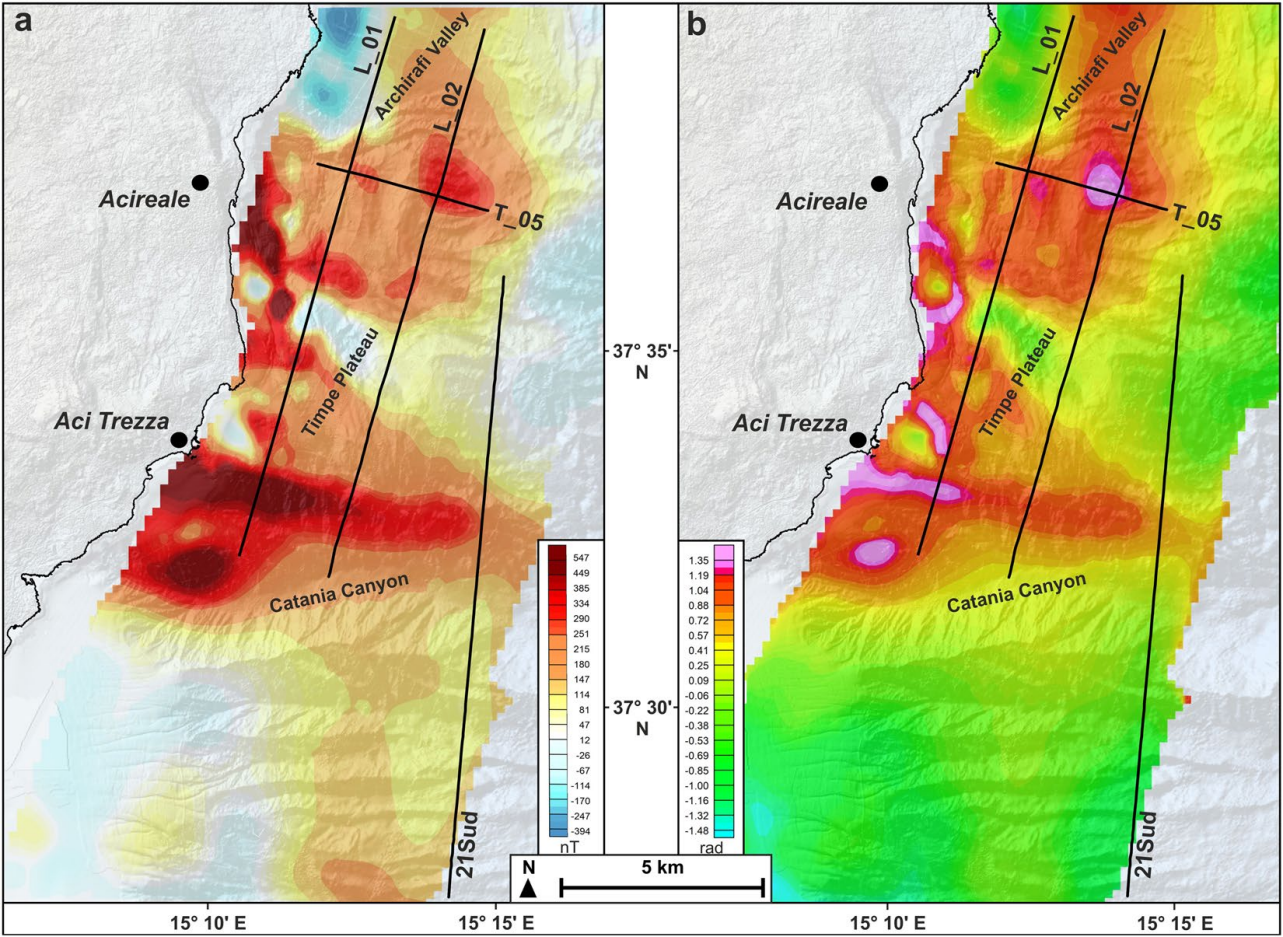
Reduced-to-the-pole (**a**) and tilt-derivative (**b**) magnetic anomaly maps of Timpe Plateau and surrounding offshore areas. Traces of seismic lines shown in this paper are reported. The prominent WNW-ESE trending positive anomaly extends offshore of Aci Trezza, suggesting a deep-seated magmatic intrusion. The presence of diffuse and scattered volcanics is highlighted by the discontinuous pattern of positive magnetic anomalies.

The Reduced-To-the-Pole (RTP) map (Fig. 2a) highlights a high-amplitude magnetic anomaly pattern showing mainly high-frequency components. The RTP field ranges from −400 to 550 nT, highlighting the high magnetic signature of the TP area, with respect to the surroundings (Fig. 2; see also^62^). The nearshore TP is characterized by scattered, very high-frequency RTP anomalies, interpreted as related to shallow intrusive bodies, which also crop out along the coast (Aci Trezza islets), and to the submarine continuation of coastal lava flows^26^. In the northern sidewall of the Catania Canyon, the highest value of the entire region indicates a main WNW-ESE elongated anomaly, which also extends onshore^63^, while a lower amplitude magnetic anomaly characterizes the distal northern TP. The tilt-angle-derivative of the pseudo-gravity map (Fig. 2b) neglects the negative regional magnetic trend related to Etna’s edifice^64^ and thus better defines the distribution of TP shallow magnetized bodies.

### Seismic reflection profiles

**Figure 3:**
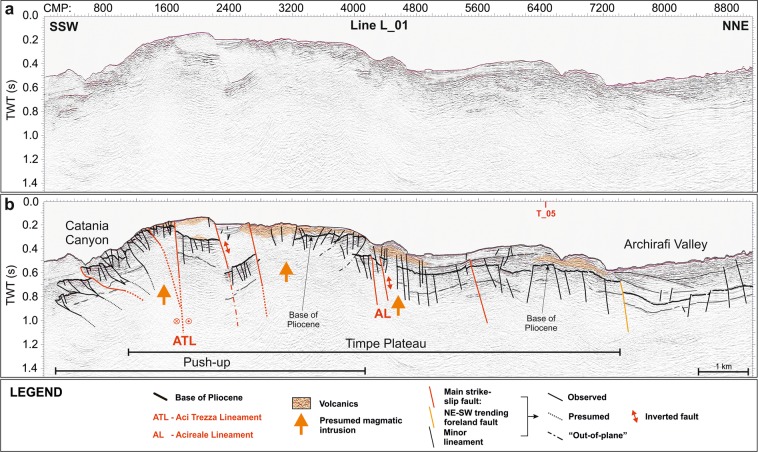
Uninterpreted (**a**) and interpreted (**b**) L_01 seismic reflection line, imaging the nearshore portion of Timpe Plateau (see Fig. 1 for location). It is a tectonically raised and highly deformed sector, rooted by a high-reflective basement, pertaining to the Hyblean foreland. The plateau is characterized by a prominent push-up in its southern portion and a bland anticline in the northern one. High-reflective chaotic and blanking seismic facies point to diffuse Plio-Quaternary volcanics (orange areas) and deep magmatic intrusions (orange arrows), respectively (see text for further details).

**Figure 4:**
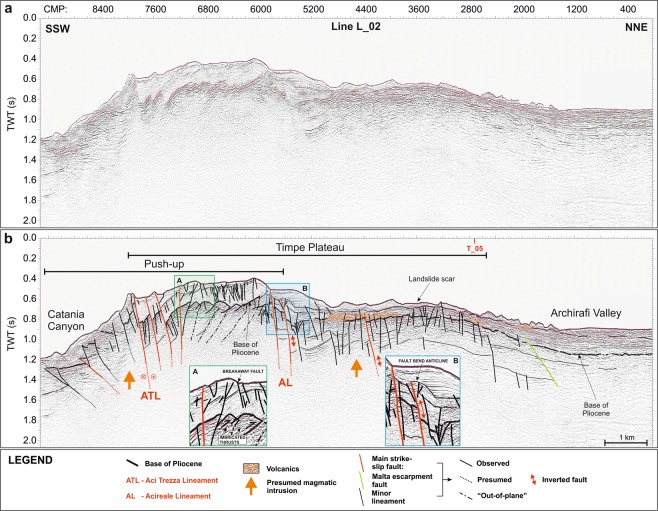
Uninterpreted (**a**) and interpreted (**b**) L_02 seismic reflection line, acquired on the distal part of Timpe Plateau (see Fig. 1 for location). The push-up is well imaged and characterized by imbricated thrusts merging into Aci Trezza Lineament (ATL). Faulting style affecting the Plio-Quaternary succession locally differs from that of the underlying substratum (Inset A). Acireale Lineament (AL) bounds the push-up to the north and locally shows evidence of positive tectonic inversion (inset B). Plio-Quaternary volcanic deposits and magmatic intrusions were detected as well (orange areas and arrows, respectively).

**Figure 5:**
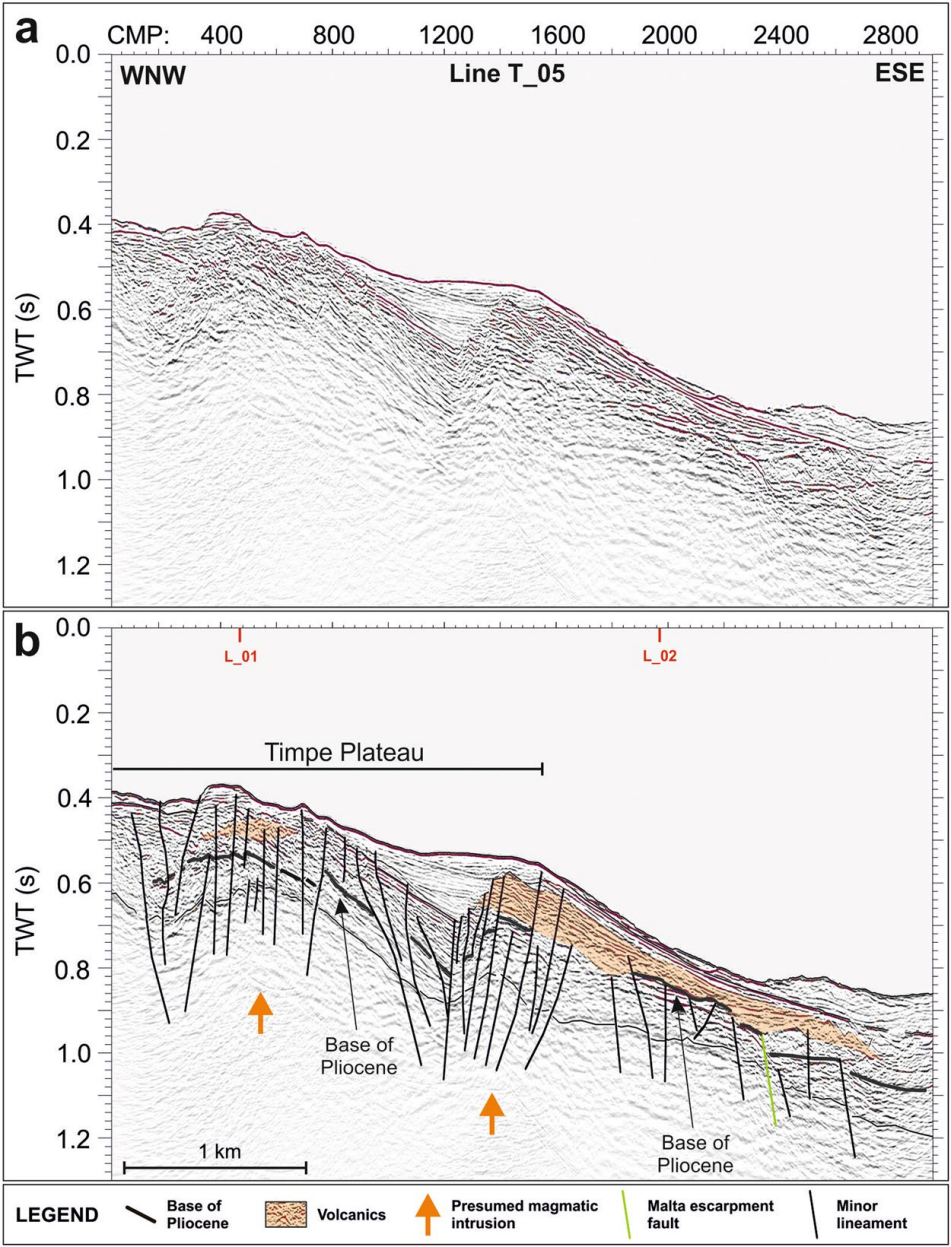
Uninterpreted (**a**) and interpreted (**b**) T_05 seismic reflection line (running perpendicularly to the L_01 and L_02 lines), which images the northern portion of Timpe Plateau (see Fig. 1 for location). Plio-Quaternary volcanic bodies (orange areas), isolating small-scale basins, and magmatic intrusions (orange arrows) were detected.

**Figure 6:**
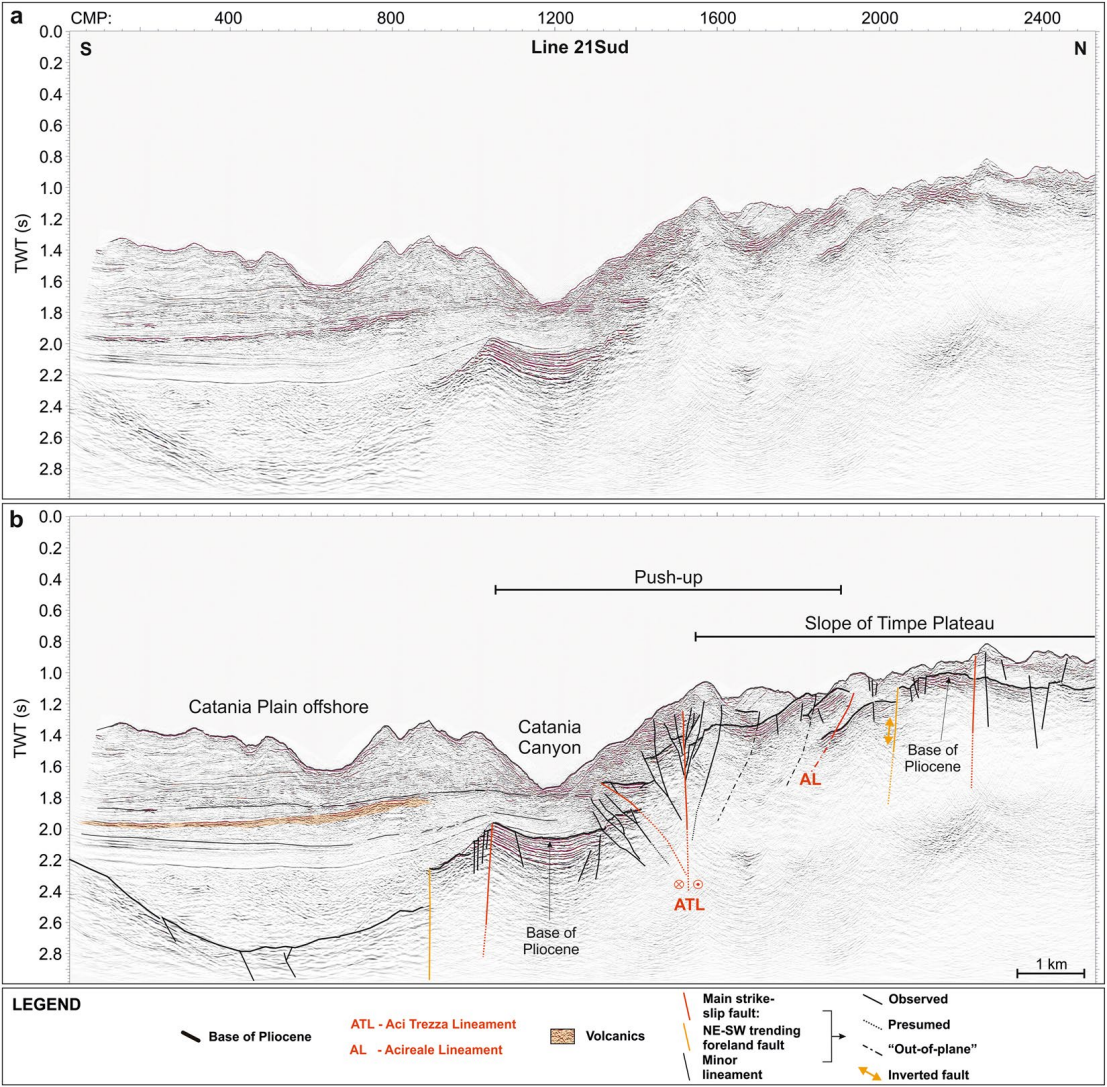
Uninterpreted (**a**) and interpreted (**b**) 21Sud seismic reflection line, cutting through the slope of Timpe Plateau and the Catania Plain offshore (see Fig. 1 for location). The latter consists of a depressed sector, rooted by the same Plio-Quaternary substratum characterizing the Timpe Plateau and filled by a thick sedimentary package with intercalated volcanics (orange areas). The push-up affects also the southeastern slope of Timpe Plateau, producing an important shortening.

High-resolution multi-channel seismic reflection profiles (Figs 3–6) reveal that the continental margin offshore of southeastern Mt. Etna is entirely rooted by a well-defined succession of highly reflective, laterally continuous and locally deformed seismic horizons, pertaining to the carbonate succession of the Hyblean foreland domain, as also found by previous studies^23,47,59^. Its top is ascribable to the base of the Pliocene sedimentary succession, mostly corresponding to the Messinian evaporites.

**Figure 7:**
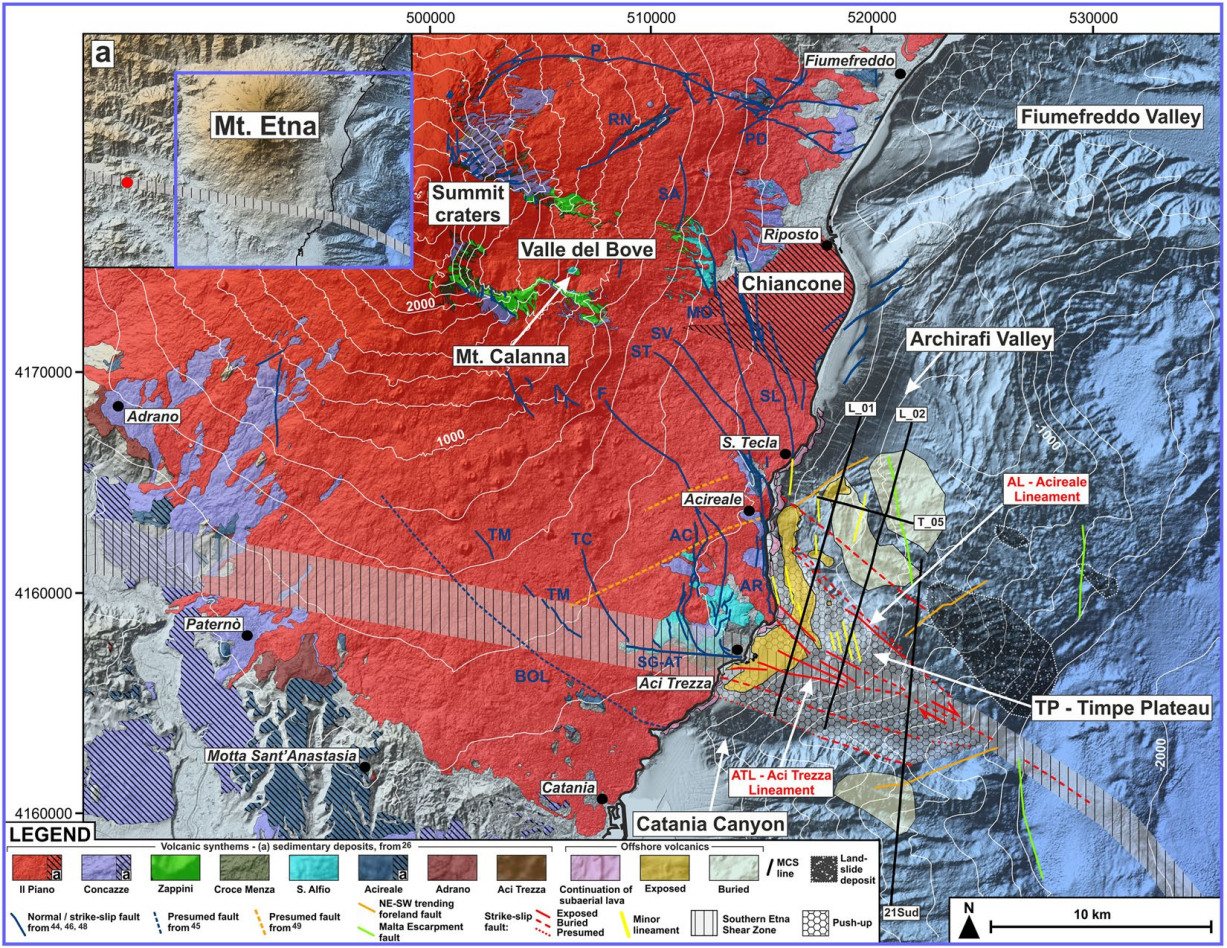
Morpho-tectonic map and volcanic features of Mt. Etna and the Timpe Plateau, delimited by inherited faults (orange and green lines) pertaining to the Hyblean domain. The wedge-shaped push-up is centred on Aci Trezza Lineament (ATL) and northwards bounded by Acireale Lineament (AL). ATL continues to the west, connecting with a regional shear zone described by ref.^17^ (red dot in Inset **a**). The tectonic arrangement of the onshore sector^45,46,48,49^ points to the subaerial continuation of the push-up (see text for details). Main offshore landslide bodies are also imaged. Main onshore faults: BOL: Belpasso-Ognina Lineament; TM: Tremestieri; TC: Trecastagni; SG-AT: San Gregorio-Aci Trezza alignment; AC: Aci Catena; AR: Acireale; F: Fiandaca; ST: S. Tecla; SV: Santa Venerina; MO: Moscarello; SL: San Leonardello; RN: Ripe della Naca; PD: Piedimonte; P: Pernicana. SA: S. Alfio Offshore faults and bathymetric data from ref.^44^ (Fig. 7 was created using the “CorelDraw X5” software).

On TP this seismic facies is recognized down to 0.8 s two-way travel time; it is exposed on the seafloor or buried beneath the Plio-Quaternary succession, imaged as an up to 0.3 s thick transparent facies, passing upwards to well-layered and laterally continuous reflectors. Along the Catania Canyon and southwards, the carbonate succession is lowered down to 2.8 s by steep faults (orange lines, Figs 6 and 7), which produce NE elongated and locally folded semi-grabens. The overlying sedimentary succession is up to 1.5 s thick and shows no evidence of tectonic deformation. Transparent and high-reflective seismic facies were also recognized (Fig. 6), ascribable to mass-wasting deposits and buried volcanic bodies, respectively.

TP is uplifted from the surrounding sectors by NNW-SSE and NE-SW oriented faults (green and orange lines respectively, Figs 3–7), conforming to the tectonic asset of the eastern Hyblean foreland^30,33^. In particular, east-dipping NNW-SSE trending lineaments, likely pertaining to the Malta Escarpment fault system, severely affect the whole continental margin, showing an en-echelon arrangement. The NE-SW faults produce a remarkable downthrow of the pre-Pliocene substratum, locally dissect the Malta Escarpment faults and show evidence of positive reactivation (Fig. 6).

The southern TP is severely offset by a WNW-ESE oriented lineament (Aci Trezza Lineament, ATL), which runs eastwards down to the bathyal plain (Fig. 7). Several WNW-ESE to NW-SE trending splays merge eastwards into ATL (red lines, Figs 3, 4, 6 and 7) and vertically displace the base of the Plio-Quaternary succession for up to 0.3 s.

The seismic images highlight the strike-slip nature of this fault system, since it produces a nearly 50 km^2^ large, wedge-shaped and roughly symmetric push-up, where the faulted blocks are laterally superimposed, producing out-of-plane reflections (Figs 3, 4, 6 and 7). Transpressive deformation has resulted in a significant shortening, with north- and south-verging imbricated thrusts, locally detached from the deeper horizons. The thrusts represent splay faults merging into ATL at depths not reached by our seismic dataset (Figs 3, 4 and 6). This tectonic structure was previously recognized along the northern sidewall of Catania Canyon^61^. The push-up is bounded to the north by the NW-SE oriented Acireale Lineament (AL, Fig. 7), which displays evidence of positive reactivation (fault-bend anticline, Fig. 4, inset B). North of the wedge, TP is only slightly deformed, as shown by the arching geometries of the reflectors (Figs 3 and 4). The southern boundary of the push-up, only partially defined by our data, seems to continue offshore of Catania, where it likely controls the sinuous path of Catania Canyon.

The deformation on TP also involves the shallow seismic horizons up to the seafloor, suggesting still active tectonics, although the faulting style affecting the Plio-Quaternary succession differs locally from that of the underlying substratum. In fact, some of the small-scale NNW-SSE trending scarps on the seafloor, sharply confined within the deformed wedge (yellow lines, Fig. 7), represent breakaway faults, produced by the gravitational failure of the sedimentary pile above the underlying en-echelon thrusts (Fig. 4, inset A).

Shallow sedimentary packages are locally affected by gravitational instability, highlighted by landslide scars (e.g. the 1.2 km wide and up to 0.7 s deep scar, Fig. 4) and associated deposits (Fig. 7).

Over the whole TP, a chaotic seismic facies topped by a highly reflective horizon, locally showing internal reflectivity and projecting acoustic shadows below, suggests the occurrence of scattered volcanic bodies (orange areas, Figs 3–5); these overlie the pre-Pliocene substratum or are interbedded within the Plio-Quaternary succession, or else are exposed at the seafloor. The volcanic nature of this seismic facies is also confirmed by positive magnetic anomalies (Fig. 2). Furthermore, the occurrence of magmatic intrusions (orange arrows in Figs 3–5), mostly localized along and between the main faults, is highlighted by seismic blanking and arching of the reflectors. On the northern sector of TP, the positive morphologies produced by the emplacement of volcanic bodies create small-scale basins, subsequently filled by up to 0.2 s Plio-Quaternary deposits (Fig. 5).

On the northern border of TP, the base of the Plio-Quaternary is lowered across NE-SW and NNW-SSE trending faults (Figs 3, 4, 5 and 7). Further north, it is covered by an over 0.3 s thick sedimentary succession, which shows no evidence of tectonic deformation (Fig. 3). Here, the Plio-Quaternary succession is imaged as high-amplitude and laterally discontinuous reflectors and is overlain by the submerged portion of the Chiancone volcanoclastic deposit^44^; this succession onlaps a seismically transparent lens, corresponding to mass-wasting bodies (Fig. 3).

Overall, it is remarkable that the tectonic pattern defined on the seismic profiles corresponds so well with the distribution of magnetic anomalies (Figs 2 and 7).

### Forward magnetic and gravity modelling

**Figure 8:**
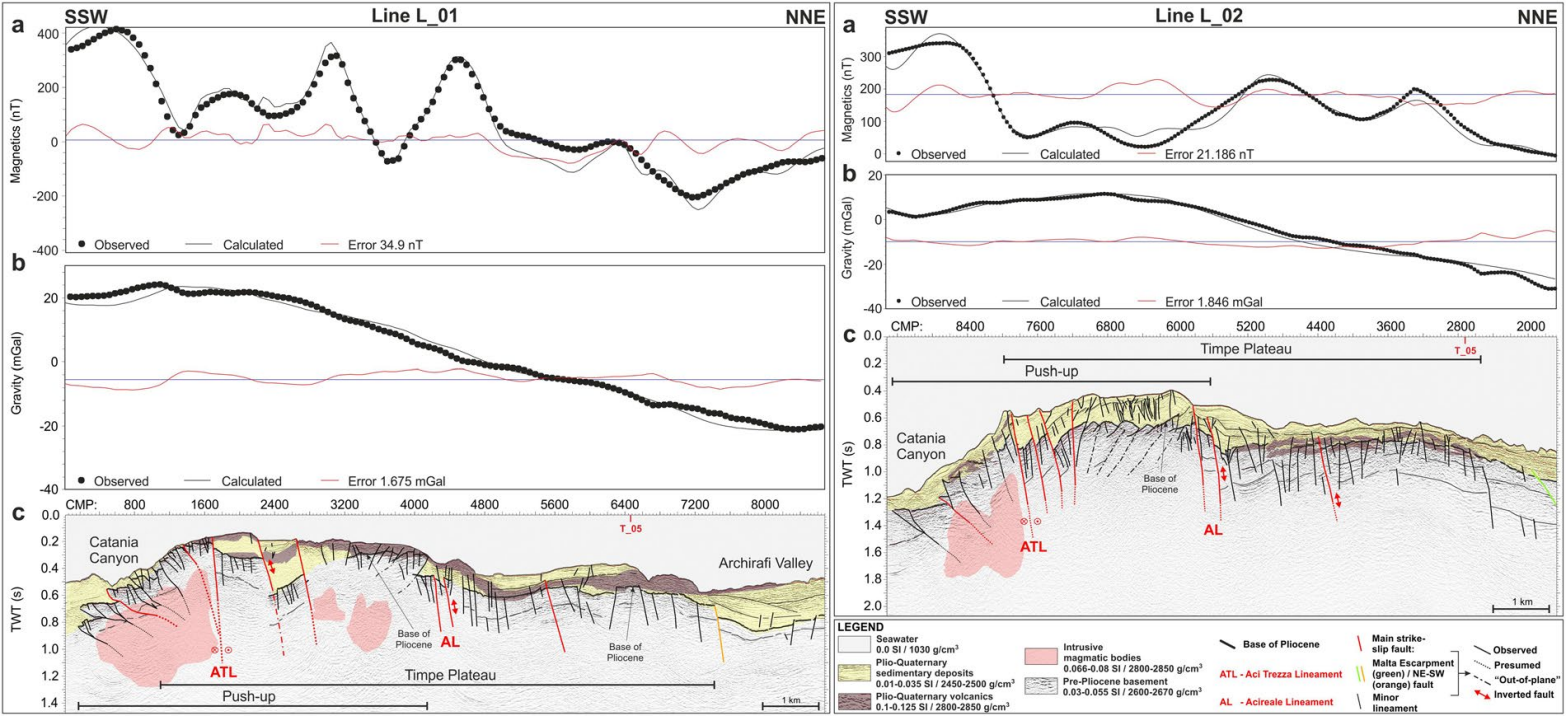
Forward magnetic and gravity modelling along the L_01 and L_02 seismic reflection lines (Figs 3 and 4). Observed, calculated and misfit (average error) curves for magnetic (**a**) and gravity (**b**) modelling. (**c**) Geometry of causative rocks, derived from time-migrated seismic sections, was used as background. The presence of a large intrusive magmatic body in the southern portion of TP is well imaged on both models.

**Figure 9:**
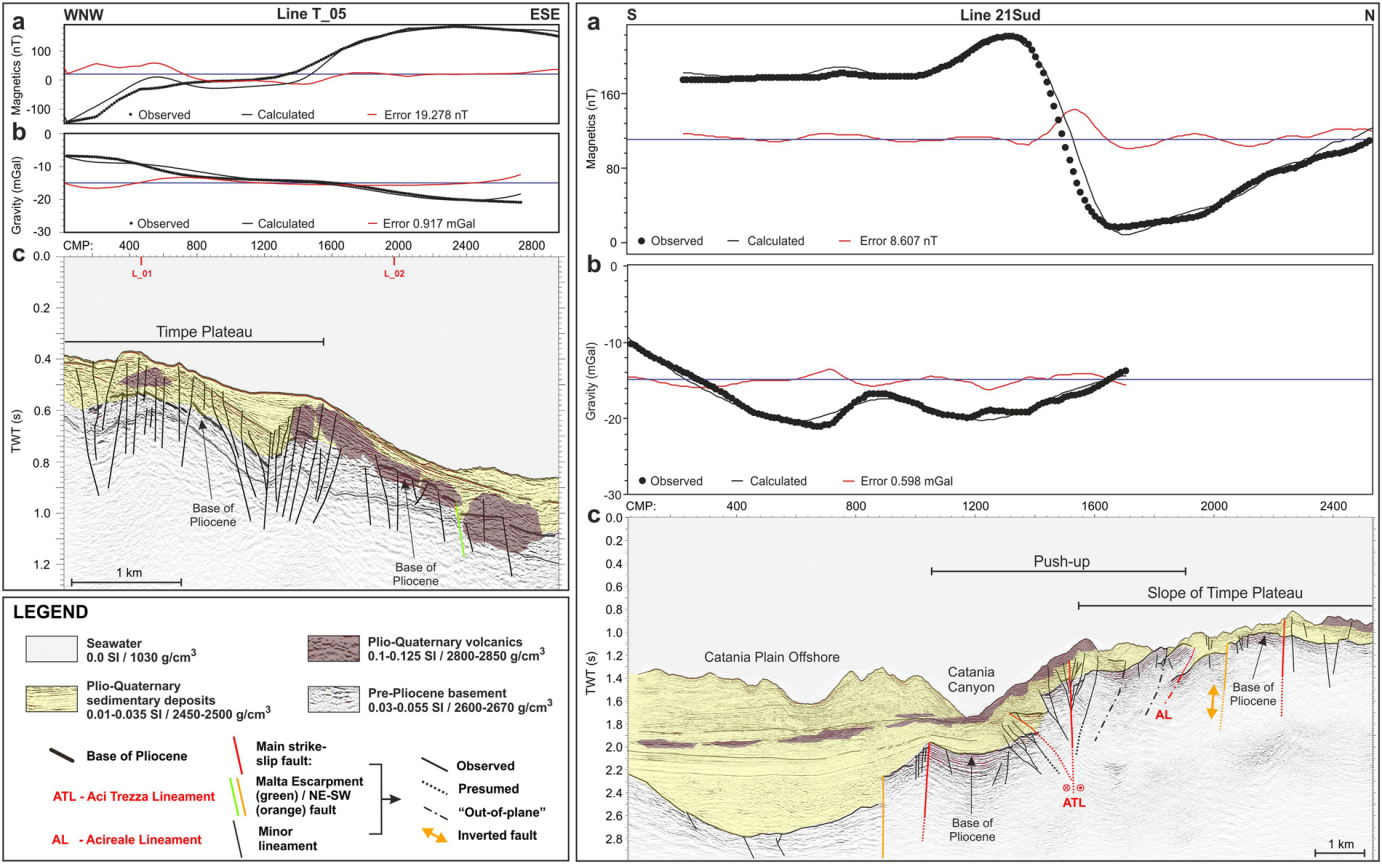
Forward magnetic and gravity modelling along the T_05 and 21Sud seismic reflection lines (Figs 5 and 6). Observed, calculated, and misfit (average error) curves for magnetic (**a**) and gravity (**b**) modelling. (**c**) Geometry of causative rocks, derived from time-migrated seismic sections, was used as background. The areal distribution and size of Plio-Quaternary volcanics are well constrained.

The 2.75D forward magnetic and gravity modelling along the seismic lines (Figs 8 and 9) validates the reliability of our seismic interpretation. All the sections confirm the presence of a thick crustal basement, as evidenced by the trend of the free-air gravity profiles (Figs 8 and 9). As expected the highest values (about 20 mGal) were recorded over the TP, while the tectonically lowered sectors of Archirafi Valley (Fig. 8, line L_01) and the offshore extension of the Catania Plain (Fig. 9, line 21Sud), are characterized by the lowest gravity values (<−20 mGal).

The depth and size of shallow and deep magmatic bodies are well-constrained. The blanking zones, detected along Catania Canyon and interpreted as deep magmatic intrusions (Figs 3 and 4), fit well with a high-density (2800–2850 g/cm^3^) and high-susceptibility body (0.066–0.08 SI, Fig. 8); frequency content is compatible with deep causative sources.

The forward models also confirm the presence of thin, high-susceptibility (0.1–0.125 SI) volcanic layers above the base of the pre-Pliocene substratum or interbedded within the Plio-Quaternary succession (see also the Catania Plain offshore), or else partially cropping out on the seafloor in the southern sector of TP (Figs 8 and 9).

## Discussion

The integrated analysis of bathymetric, high-resolution seismic reflection, magnetic and gravity data, allowed us to characterize in detail the crustal structure offshore of the southeastern slope of Mt. Etna, which represents a pivotal area to understand the spatial and temporal evolution of volcanism in this region.

The nearshore of TP (depths < 40 m) is floored by the submarine continuation of the coastal lava flows pertaining to the last 200 ka volcanic activity^26^ (pink areas, Fig. 7). Besides these lava flows and the volcanic islets offshore of Aci Trezza, TP and surroundings show evidence of volcanics exposed on the seafloor or buried beneath a shallow sedimentary coverage (yellow areas, Fig. 7). Scattered volcanic deposits, laying directly on top of the pre-Pliocene substratum or interbedded within the Plio-Quaternary succession, were also detected (light-yellow areas, Fig. 7). The volcanic bodies on TP cover an overall area of about 40 km^2^ and are locally associated with magmatic intrusions along or between the main faults (Figs 7–9); the seismo-stratigraphic position, as well as location of volcanic bodies, suggest an older or almost coeval age with the first phases of Etna volcanism ( > 120 ka).

The scattered distribution of volcanic bodies does not uniquely determine the morphology of the TP (as inferred by ref.^44^), which instead represents, on the basis of our results, an inherited rhombohedral-shaped horst pertaining to the carbonate succession of the Hyblean domain. The TP horst is raised by the Malta Escarpment and the NE-SW trending fault segments (respectively green and orange lines in Figs 3–9). Furthermore, a WNW-ESE to NW-SE trending strike-slip fault system (red lines in Figs 3, 4, 6–9) dissects the TP and relative deeper areas down to the bathyal plain. It pertains to the South-Tyrrhenian System^36^, which, since the Tortonian, accommodated the dextral displacement of the major tectonic domains in Sicily, as a consequence of the spreading of the back-arc Tyrrhenian Basin^18,24^. Successively, its kinematics was mainly controlled by the NW-SE oriented regional maximum horizontal stress axis^32,34^, related to the northwestward moving Hyblean block (Fig. 1a).

On the TP, the strike-slip fault system is centred on the WNW-ESE trending ATL and severely deforms the pre-Pliocene substratum, producing a symmetric wedge-shaped push-up, delimited to the north by AL (Fig. 7); the transpressive deformation further lifts the pre-existing TP morpho-structural high, also inverting its inherited Hyblean distensive structures, which delimit the TP high (green and orange lines, Figs 3–9). Besides the above evidence from the seismic images, the strike-slip kinematics of ATL is also highlighted by the dextral displacement of the volcanic bodies offshore of Aci Trezza^44^ and by submarine geodetic data^65^. Furthermore, ATL likely extends eastwards, down to the bathyal plain, presumably connecting with a regional-scale dextral transcurrent lineament, previously identified by several authors^7,12,44,59,61^ and interpreted as a STEP fault^38,66^. Transpressive deformation could have also favoured the gravitational instability affecting the TP eastern margin, as highlighted by a remarkable landslide scar (Fig. 4), associated to a large deposit (Fig. 7).

The tectonic arrangement of the onshore sector points out the subaerial continuation of the strike-slip related deformation: the San Gregorio-Aci Trezza alignment (SG-AT, Fig. 7) represents the surficial expression of ATL, while the NW-SE trending S. Tecla and S. Venerina faults (ST and SV, Fig. 7) extends landward roughly from AL. Instead, the presumed NE-SW trending faults^49^ (dotted orange lines, Fig. 7) appear as the onshore continuation of those bounding TP (orange lines, Figs 3, 6–9).

Southwest of Mt. Etna, along the westward continuation of the ATL-San Gregorio-Aci Trezza alignment, a regional-scale shear zone (red dot, Fig. 7a) was identified on a nearly N-S section crossing Sicily, compiled on the basis of geological and geophysical data^17^. Here, the shear zone interrupts the continuity of the Hyblean basement and of the overlying Apenninic-Maghrebian thrust-sheet system and also produces an abrupt discontinuity in the magnetic basement. Moreover, other authors^19,20^ inferred the existence of post-Tortonian regional dextral transcurrent faults in adjacent areas, also controlling the evolution of local Messinian and Pliocene depocenters. Dextral strike-slip tectonics along WNW-ESE fault planes is also consistent with the main NW oriented maximum horizontal stress axis characterizing the region^32,34^, as also confirmed by the focal mechanisms solutions of recent earthquakes^67^. This evidence points to the occurrence of a regional WNW-ESE trending dextral main shear zone (hereby-named “Southern Etna Shear Zone”, Fig. 7) running south of Mt. Etna and extending offshore along ATL.

Compression and related uplift in the coastal sector of TP^54,57^ are the direct effect of the transpressive deformation induced by the north- and south-verging thrusts of the roughly symmetrical strike-slip related push-up (Figs 3, 4, 6–9), developing along ATL. Analogously, we argue that the compression observed between Catania and Motta Sant’Anastasia is induced by the “Southern Etna Shear Zone” kinematics and therefore not strictly by the active frontal folds of the Apenninic-Maghrebian chain^55–57^, or by the Etna edifice gravitational spreading^50,51^. The lack of allochthonous seismo-stratigraphic units offshore of southeastern Etna strongly supports our inference, suggesting also that the most advanced allochthonous nappes of the collisional belt are located further to the northwest, beneath the volcanic pile, as also proposed by ref.^68^.

### Neo-tectonics of Mt. Etna

Strike-slip related deformation also locally controls neo-tectonic features in the Mt. Etna region, both off and onshore. On TP, faulting style affecting the Plio-Quaternary succession is often decoupled from that of the underlying substratum, as evidenced by some NNW-SSE trending breakaway faults, produced by the gravitational failure of the shallow sedimentary pile above the transpressive structures (Fig. 4 and yellow lines within the push-up on TP, Fig. 7). Similarly, the sub-parallel onshore Acireale and Aci Catena faults (AR and AC, Fig. 7) and relative minor structures appear as the surficial effect of the volcanic pile creeping failure and coseismic rupture above push-up splays. This hypothesis explains the presence of prominent and active extensional structures^46^ in an area affected by important shortening and the westward back-tiling of the blocks confined to the east by AR and AC^6,46,69^; our interpretation is further validated because AR, AC, and sub-parallel offshore fault segments represent local structures, being sharply confined within ATL and AL and related onshore continuations. Therefore, on the basis of our observations, the same push-up affecting the TP continues onshore at least up to the AC fault (Fig. 7).

Conversely, faults extending north of S. Tecla (e.g. SL and MO, Fig. 7) are likely not involved in the same kinematics since they affect an area outside of the push-up and characterized by remarkable subsidence^43,54^. All the above-described fault segments, together with others (e.g. P, TM, TC, F and BOL, Fig. 7), represent preferential shallow structures locally conditioning Mt. Etna’s eastern flank seaward sliding. This movement is less pronounced in the southeastern sector of the volcano up to the onshore continuation of AL (i.e. ST and SV faults^43,45^, Fig. 7), because it is hampered by the transpressive deformation, further lifting the pre-existing TP morpho-structural high (and its onshore continuation, Fig. 7). In contrast, flank sliding is enhanced to the north, probably favoured by the presence of a tectonically lowered sector (Archirafi Valley, Fig. 7). Therefore, seaward gravitational sliding of the Etna eastern flank superimposes on the pre-existing morpho-structural setting of the substratum, even though is also triggered by magmatic intrusions.

Some of the afore-mentioned faults have produced important earthquakes, including recent ones (e.g. the Mw 4.9, 26/12/2018 event along the dextral transtensive Fiandaca fault; http://cnt.rm.ingv.it/event/21285011/?tab = MeccanismoFocale#TDMTinfo).

### Evolutional model of Etna volcanism

**Figure 10:**
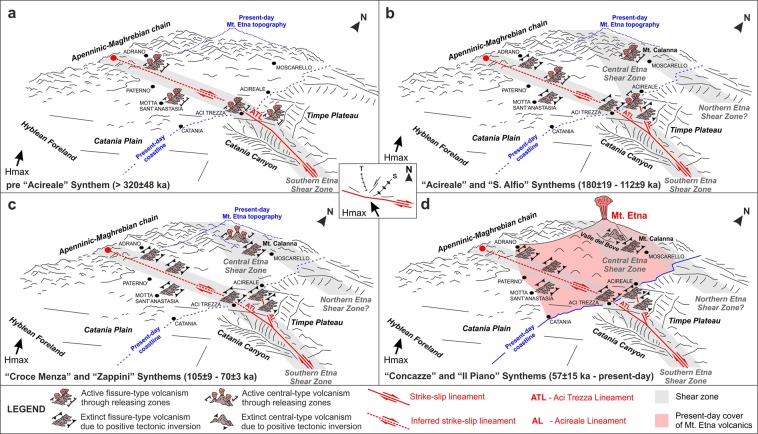
Schematic conceptual model illustrating the time and space evolution of volcanism in the Mt. Etna area driven by strike-slip tectonics, which is controlled by the regional stress field shown in the central inset (T: Tension; S: Shortening; Hmax: Horizontal maximum stress axis; modified from ref.^19^). Volcanic Synthems from ref.^26^. Panel (a) The WNW-ESE trending dextral “Southern Etna Shear Zone”, offshore centred on ATL and continuing onshore up to the regional transcurrent lineament described by ref.^17^ (red dot), produced scattered and fissure-type volcanism at local releasing zones, both off and onshore, from the TP area to the surroundings of Adrano. Panel b - Following the model by refs^15,16^, migration of releasing and restraining regions along the “Southern Etna Shear Zone” favoured new fissure-type volcanism and hampered the former one, with positive tectonic inversion affecting the previous releasing zones (e.g. the push-up observed on TP). Meanwhile, releasing zones migrated northwards along the NW-SE trending “Central Etna Shear Zone”, of which the offshore expression results in AL, favouring volcanism along the Acireale coast up to Moscarello and Mt. Calanna. This second shear zone may represent a splay fault of ATL or a step-over belt between the “Southern Etna Shear Zone” and an analogous dextral sub-parallel structure to the north (a possible “Northern Etna Shear Zone”). Volcanism along the “Southern Etna Shear Zone” ceased (**c**); a further migration of releasing and restraining zones produced a new shifting of volcanism firstly to the present-day Valle del Bove area (**c**) and then to the Mt. Etna summit craters (**d**), (see text for further details).

Although several studies reported on volcanic activity associated with transpressional tectonics^14^, the current deformation of TP apparently does not fit with local volcanism, as magmatic processes are usually favoured by a tensional regime. To resolve this ambiguity and explain the rapid northward shifting of Etna’s eruptive vents, we propose a comprehensive conceptual model (Fig. 10) linking the time-space evolution of volcanism with strike-slip tectonics.

Since at least 500 ka up to about 320 ka (*pre-Acireale Synthem*^25,26^), wrench tectonics along the WNW-ESE trending “Southern Etna Shear Zone” favoured scattered fissure-type volcanism through releasing zones, both off and onshore, from the TP area, through Aci Trezza, Motta Sant’Anastasia and Paternò, up to Adrano (Fig. 10a); indeed, the deeper magmatic intrusions run strictly along the ATL (Figs 2 and 8). Localized lithospheric stretching, eventually also occurring through the reactivation of inherited Hyblean structures, produced volcanism at releasing regions, controlled by the main NW oriented horizontal maximum stress axis (central inset, Fig. 10, modified from ref.^19^). Under this tectonic regime, the Riedel model for dextral simple shear predicts a roughly ENE-WSW oriented maximum extension, in agreement with the occurrence of perpendicular^70^, roughly NNW-SSE oriented eruptive fissures, characterizing the volcanism up to the S. Alfio Synthem^25,26^.

According to the model proposed by Wakabayashi^15,16^, the continuing strike-slip deformation led to the migration of releasing and restraining regions along the same “Southern Etna Shear Zone”, favouring the opening of new eruptive fissures and hampering the former ones (Fig. 10b). Thus, the previous releasing zones were affected by a positive tectonic inversion, which produced transpressive structures as the push-up observed on TP. Releasing zones migrated also northwards, presumably along a second shear zone (hereby-named “Central Etna Shear Zone”, NW-SE oriented, extending onshore from AL), favouring magma ascent along the Acireale coast up to Moscarello and Mt. Calanna (*Acireale* and *S*. *Alfio Synthems*, 180 ± 19–112 ± 9 ka^25,26^). At the present, the lack of comparable information on the northern sector of Mt. Etna and related offshore areas does not allow us to assert whether this second shear zone is a splay fault of ATL, or a step-over belt between the “Southern Etna Shear Zone” and an analogous dextral sub-parallel structure to the north (a possible “Northern Etna Shear Zone”).

At about 105 ka, volcanism along the “Southern Etna Shear Zone” definitively ceased (Fig. 10c), likely also due to the ongoing indentation of the northwestward moving Hyblean block^71^. Thus, continuing deformation led not only to prevailing transpressive kinematics along the whole “Southern Etna Shear Zone” (hampering magma ascent) but also to the activation of other shear zones to the north. The development and subsequent migration of releasing and restraining regions along the “Central Etna Shear Zone” produced a new shifting of volcanism, first to the present-day Valle del Bove area, where it evolved from fissure- to central-type (*Croce Menza* and *Zappini Synthems*, 105 ± 9–70 ± 3 ka^25,26^, Fig. 10c), and then to the Mt. Etna summit craters (*Concazze* and *Il Piano Synthems*, 57 ± 15 ka - present-day^25,26^, Fig. 10d).

Migration and recent positive tectonic inversion of releasing regions are strongly suggested by fault-bend anticlines deforming the Plio-Quaternary basinal sediments offshore of southeastern Mt. Etna. This process explains the unexpected compressive structures affecting the Motta Sant’Anastasia^50,51^ and Mt. Calanna^72^ areas, which were the sites of a roughly 320 ka and 130 ka old volcanism, respectively.

The whole Etna area is affected by a wide wrench region, characterized by different shear zones. Their activity and mutual interaction led to local transtension and subsidence (which favoured volcanism), rapidly evolving to transpression and uplift (which hampered volcanism), resulting in the overall time-space scattering of the volcanic centres^25^ (Fig. 10). The proposed reconstruction provides a useful key to understanding the temporal and spatial spreading of the eruptive centres of Mt. Etna, the largest and most active volcano in Europe, and one of the most studied worldwide. The model also sheds new light on the nature of the tectonic deformation heavily affecting the southeastern coastal sector of the volcano, with both seismicity and flank movement.

Our findings also advance the wider understanding of the interaction between tectonics and volcanism, providing a useful key to explain volcanism in analogous contexts worldwide.

## Methods

### Multibeam data

High-resolution morpho-bathymetric maps were obtained using shallow (455 kHz Reson Seabat) and deep-water (50 kHz Reson Seabat and 70–100 kHz Kongsberg) multibeam sonar systems, employed during several research cruises between 2005 and 2009^44^. In particular, a very high resolution (1 m cell-size) marine digital terrain model was obtained in shallow water down to 120 m bsl, while a 20 m gridded one was obtained down to about 2000 m water depth. “Hips & Sips - Teledyne CARIS” software was used for data processing. The deepest sectors were imaged using the bathymetric data products derived from the “EMODnet Bathymetry portal” (http://www.emodnet-bathymetry.eu).

### Magnetic and gravimetric data

Nearly 2000 km of high resolution shipborne magnetic data were recorded during the TOMO-ETNA experiment in 2014–2015 using a Geometric G881 caesium-pumped magnetometer, towed 180 m astern of the vessel^62,73^. Raw magnetic data were collected at 1 Hz sampling frequency and processed applying a band-pass filter, despiking, diurnal correction, and statistical leveling. Total intensity magnetic anomaly field was computed by removing the International Geomagnetic Reference Field^74^. A reduced-to-the-pole anomaly map was constructed by applying a phase shift transformation in FFT domain, which minimizes the dipolar behaviour of Earth’s magnetic fields^75^, allowing a direct relationship between magnetic anomalies and causative sources. To avoid residual negative values in the RTP map (Fig. 2) related to an imperfect minimization of the regional contribution of the Mt. Etna edifice, an additional interpretative approach was performed by computing the tilt-angle-derivative of pseudo-gravity distribution^76^ (Fig. 2). This process is based on the ratio between the first vertical derivative and horizontal gradient magnitude^77,78^ and does not change the spectral content of data.

Gravity data were acquired during the same TOMO-ETNA experiment using an Air/Sea Micro-g LaCoste dynamic gravity meter. Raw gravity data were sampled at 1 Hz interval, smoothed using a low-pass (120 s) filter and corrected for cross-coupling errors and the Eotvos effect. Relative gravity readings were tied to an absolute gravity station located at Syracuse harbour. Instrumental drift was also estimated and subtracted from the gravity data by using a linear trend. Latitude correction was applied to obtain the free air anomaly field.

Joint interpretation of magnetic and gravity potential fields was achieved by applying a 2.75D forward modelling along the seismic lines (Figs 8 and 9). The magnetic anomaly and free-air gravity profiles were sampled from interpolated grids with a cell size of 250 m. The model was constrained by geometries of crustal bodies identified through the seismo-stratigraphic interpretation of seismic sections. The forward model was then constrained in-depth assuming reliable seismic velocities; these last were converted to density values by using Gardner’s Equation^79^, which provided the starting benchmark for the subsequent adjustments. Adding the information from the seismic stratigraphy, we minimized the ambiguity of the results, better constraining the modelling approach.

### Seismic reflection data

The seismo-stratigraphic and structural framework of the area was reconstructed using a close-spaced grid of high-resolution multi-channel seismic reflection profiles, acquired in 2014, during the marine activities of the TOMO-ETNA experiment^73^. These data were processed, using the “VISTA desktop seismic data processing” software by Schlumberger, at the “Istituto Nazionale di Geofisica e Vulcanologia (INGV) - Osservatorio Etneo” (Catania, Italy) in collaboration with the “Istituto Nazionale di Oceanografia e Geofisica Sperimentale” (Trieste, Italy). Acquisition methodologies and main processing steps are reported in^80^; due to the complexity of the area, additional procedures were applied to further increase the signal-to-noise ratio. The dataset was integrated with other seismic profiles acquired offshore of Mt. Etna by the INGV in 2005, made available through the “SOME” (Seismic lines Offshore Mount Etna) open database^81^ and other interpreted seismic databases^44,59,61^. Seismic profiles show an overall good quality, with an estimated vertical resolution of 2–3 m in the shallower sedimentary succession, while most of the energy is hardly able to penetrate a high-impedance substratum, identified down to about 3 s two-way travel time.

To image the shallower portions of the study area with higher resolution, particularly to identify fault-induced displacements, volcanic and slump deposits at the seafloor or beneath few tens of meters of soft sediments, a dense grid of sparker profiles were interpreted (Fig. 1). All processed data were imported into the “Kingdom” software by HIS-Markit for interpretation.

## Data Availability

The datasets analysed during the current study are available from the corresponding author on reasonable request.
